# Predicting Intensive Care Unit Length of Stay After Acute Type A Aortic Dissection Surgery Using Machine Learning

**DOI:** 10.3389/fcvm.2021.675431

**Published:** 2021-07-12

**Authors:** Qiuying Chen, Bin Zhang, Jue Yang, Xiaokai Mo, Lu Zhang, Minmin Li, Zhuozhi Chen, Jin Fang, Fei Wang, Wenhui Huang, Ruixin Fan, Shuixing Zhang

**Affiliations:** ^1^Department of Radiology, the First Affiliated Hospital, Jinan University, Guangzhou, China; ^2^Graduate College, Jinan University, Guangzhou, China; ^3^Department of Cardiac Surgery, Guangdong Cardiovascular Institute, Guangdong Provincial People's Hospital, Guangdong Academy of Medical Sciences, Guangzhou, China

**Keywords:** acute type A aortic dissection, surgery, intensive care unit, length of stay, machine learning

## Abstract

**Background:** Patients with acute type A aortic dissection are usually transferred to the intensive care unit (ICU) after surgery. Prolonged ICU length of stay (ICU-LOS) is associated with higher level of care and higher mortality. We aimed to develop and validate machine learning models for predicting ICU-LOS after acute type A aortic dissection surgery.

**Methods:** A total of 353 patients with acute type A aortic dissection transferred to ICU after surgery from September 2016 to August 2019 were included. The patients were randomly divided into the training dataset (70%) and the validation dataset (30%). Eighty-four preoperative and intraoperative factors were collected for each patient. ICU-LOS was divided into four intervals (<4, 4–7, 7–10, and >10 days) according to interquartile range. Kendall correlation coefficient was used to identify factors associated with ICU-LOS. Five classic classifiers, Naive Bayes, Linear Regression, Decision Tree, Random Forest, and Gradient Boosting Decision Tree, were developed to predict ICU-LOS. Area under the curve (AUC) was used to evaluate the models' performance.

**Results:** The mean age of patients was 51.0 ± 10.9 years and 307 (87.0%) were males. Twelve predictors were identified for ICU-LOS, namely, D-dimer, serum creatinine, lactate dehydrogenase, cardiopulmonary bypass time, fasting blood glucose, white blood cell count, surgical time, aortic cross-clamping time, with Marfan's syndrome, without Marfan's syndrome, without aortic aneurysm, and platelet count. Random Forest yielded the highest performance, with an AUC of 0.991 (95% confidence interval [CI]: 0.978–1.000) and 0.837 (95% CI: 0.766–0.908) in the training and validation datasets, respectively.

**Conclusions:** Machine learning has the potential to predict ICU-LOS for acute type A aortic dissection. This tool could improve the management of ICU resources and patient-throughput planning, and allow better communication with patients and their families.

## Introduction

Acute type A aortic dissection is one of the leading causes of mortality worldwide, with a spontaneous mortality of 1–3% per hour within the first 48 h (1). As the mortality rate is very high, immediate surgery is indicated. After surgery, medical care provided to patients in the intensive care unit (ICU) is labor intensive and costly (2). The ICU-length of stay (ICU-LOS) of patients varies substantially. Accurate prediction of ICU-LOS is of great significance in acute type A aortic dissection, especially in the context of an aging population and increasing cardiovascular surgeries. It is one of the effective solutions to tackle capacity management, recourse planning, and staffing levels (3–5).

Although there were models for predicting ICU-LOS, they had relied on conventional statistical methods, which might limit their application and performance in a larger dataset with multiple variables and samples (6–12). Recently, computational methods such as machine learning have attracted more and more attention due to their ability to predict events occurrence and aid in clinical decision-making (13). Machine learning refers to a body of methods based on computer science that use patterns in data to identify or predict an outcome. It provides a powerful set of tools to describe association between the features and outcomes of interest, particularly when they are nonlinear and complex (14–16). It is best used when there are huge number of variables, and overfitting (poor generalizability) can be a problem for traditional statistical methods. Accordingly, our aim was to design and evaluate supervised machine learning models to predict ICU-LOS based on preoperative and intraoperative data of patients after type A aortic dissection surgery.

## Materials and Methods

### Patients and Data Sources

This retrospective study was approved by our institutional review board, and the informed consent from patients was waived. The entire cohort was patients who were diagnosed with acute type A aortic dissection (17) between September 2016 and August 2019 in Guangdong Provincial Cardiovascular Institute. All the patients were confirmed by computed tomography or transesophageal echocardiography. After acute type A aortic dissection surgery, the patients were transferred to ICU immediately. Patients were characterized by 84 readily available preoperative (including demographics, clinical manifestations, medication history, previous history, vital signs, laboratory findings, and auxiliary examinations) and intraoperative (including surgical types, surgical times, surgical technique, and intraoperative observation) variables. Data were input by experienced physicians and nurses and each record was audited by dedicated trained technical and medical teams. For the classification of dissection aneurysm, we used a modified version of the Stanford types proposed by Beijing Anzhen Hospital, Capital Medical University. Stanford's type A is classified as type C and type S according to the lesion of the aortic arch. Type C is defined as one of the following: (1) the primary intimal tear locates in aortic arch or distal aorta, and the dissection retrogrades to the ascending aorta or proximal aortic arch; (2) aortic aneurysm exists in aortic arch or distal aorta (diameter > 5 cm); (3) the involvement of brachiocephalic artery; and (4) caused by Marfan's syndrome. Type S is defined as follows: the location of the primary intimal tear is in the ascending aorta, without any lesions of type C.

### Feature Selection for Modeling

Feature selection is an essential but important process of building a machine learning model. It implies some degree of cardinality reduction by reducing the number of features used to build a model. In this study, features with missing values more than 20% were excluded. There were a large number of variables after data cleaning, sampling, and preprocessing. Thus, we used Kendall rank correlation coefficient to select the significant features. The features with Kendall's tau ranked in the top 25% were identified.

### Models' Development, Evaluation, and Validation

We divided the ICU-LOS into four intervals (<4, 4–7, 7–10, and >10 days) according to its interquartile range. Five classic machine learning models with five-fold cross-validation were developed to predict ICU-LOS, namely, Naive Bayes (NB), Linear Regression (LR), Decision Tree (DT), Random Forest (RF), and Gradient Boosting Decision Tree (GBDT). Overall, the original dataset was randomly split into a training (247 cases, 70%) dataset and a held-out validation (106 cases, 30%) dataset. Classification performance of the machine learning models was measured using the area under the curve (AUC) and the associated 95% confidence interval (CI), which was subjected by bootstrapping 100 times. Machine learning models were implemented in open-source Python 3X and Project Jupyter version 1.2.3 (Anaconda, Inc., https://jupyter.org/about). The descriptions of machine learning models were shown below.

#### Naive Bayes

Based on Bayes theorem, NB is a probabilistic classifier with a strong assumption of independence among variables or features. It has solid mathematical foundation and stable classification efficacy. It requires few parameters to estimate and it is not sensitive to missing data. The algorithm is relatively simple, with a small error rate. The classification principle is based on the prior probability of an object, using Bayes formula to calculate the posterior probability, that is, the probability that each object belongs to a specific class. The class with the maximum posterior probability is selected as the class that the object belongs to.

#### Linear Regression

LR is a kind of generalized linear regression algorithm. The independent variables of the LR model can accept a wide range of data types, including continuous and discrete variables. The LR model is easy to be trained and its parameters are easy to be explained, so it is widely used in the biomedical field, especially in epidemiology. This model uses a sigmoid function to predict the logistic transformation of the probability for each class in the dependent variable. The logged odds classify the data points in a binary fashion. The lambda parameter used for the model was a ridge value of 1.0E−8 in addition to conjugate gradient descent. Conjugate gradient descent was applied to reduce the cost function in the model. Basically, in the case of classification, the learned LR classifier is actually a set of weights θ. When there is test sample input, the weights and test data are weighted. The formula of LR is shown below.

(1) $$\begin{array}{r} P\left(y=1|x;\theta \right)=\frac{1}{1+e^{-}\theta^{Tx}} \end{array}$$

#### Decision Tree

There are many advantages to use the DT model in classification problems, such as low computational complexity, convenience, and efficiency. It can process data with unrelated characteristics and construct rules that are easy to be explained and understood. DT consists of nodes and directed edges. There are two types of nodes: the internal node, which represents a feature or attribute, and the leaf node, which represents a class. In general, a DT contains a root node, several internal nodes, and several leaf nodes. DT can be thought of as a collection of if–else rules. A rule is constructed from each path from root node to leaf node. The feature of inner node corresponds to the condition of the rule, and the leaf node corresponds to the decision result of the rule. The paths of DT are mutually exclusive but complete; that is, each instance is covered by only one path or one rule. The purpose of DT classifier learning is to produce a decision tree with strong generalization ability to deal with the unseen examples.

#### Random Forest

RF is an ensemble learning algorithm based on DT. It is very simple, is easy to implement, and has very little computing overhead, but shows amazing performance in classification and regression. Therefore, RF is praised as a method representing the technology level of ensemble learning. RF applies an ensemble of DT and bootstrapping to sample training data and split branches in each tree. The target in each split is to maximize the gained information from each random feature in each sample per tree. After evaluating the data points, the resulting class is the mode of the results of all trees. Briefly, each DT is a classifier, so for an input sample, N trees will have N classification results. The RF integrates all the classified voting results and specifies the classification with the most votes as the final output.

#### Gradient Boosting Decision Tree

The working mechanism of the Boosting algorithm is to train a weak learner 1 with the initial weight of the training dataset, and update the weight of the training sample according to the learning error rate of the weak learner, so that the weight of the training sample points with high learning error rate in the previous weak learner 1 becomes higher. Then, these points with high error rate get more attention in weak learner 2, and the training set with the adjusted weight is used to train weak learner 2. This is repeated until the number of weak learners reaches the pre-specified number T, and the T weak learners are integrated through the set strategy to get the final strong learner. After knowing the Boosting method, we can combine the Boosting method with the DT to get the GBDT.

### Statistical Analysis

All statistical analyses were performed with Python 3.X and Project Jupyter 1.2.3 (Anaconda, Inc, https://jupyter.org/about). The packages were used as follows: “fitcnb” for NB, “glmfit” for LR, “DecisionTreeClassifier” for DT, “TreeBagger” for RF, and “GradientBoostingClassifier” for GBDT. Missing data were assumed to be missing at random and were imputed using 10-fold multiple imputation by chained equations. Continuous variables were expressed as mean ± standard deviation, while categorical variables were expressed as counts (percentages) of the total population. Comparisons were considered statistically significant based on a two-sided *p*-value of <0.05.

## Results

### Patient Characteristics

A total of 353 patients (307 males and 46 females; mean age of 51.0 ± 10.9 years) transferred to ICU after acute type A aortic dissection surgery was included. These patients were randomly assigned to a training dataset (*n* = 247) and a validation dataset (*n* = 106). The median ICU-LOS of the patients was 7.7 days (interquartile range, 4.8–11.9 days; range, 0.2–70.5 days). Four patients died after 2.9, 5.0, 19.0, and 44.4 days after ICU admission, respectively. Two patients died of septic shock, one died of extensive bleeding due to coagulation disorders, and one died of multiple organ dysfunction syndrome. Except for aortic dissection surgery, the four patients also received hemodialysis therapy and one of them required tracheotomy.

The initial predictor variables included 84 preoperative and intraoperative features for each patient. After data cleaning, sampling, and preprocessing, 11 variables with low correlation were excluded, and 73 variables (58 preoperative features and 15 intraoperative features) were finally included in the analysis. Supplementary Table 1 shows the comparisons of baseline characteristics between the training and validation datasets. All characteristics except respiratory frequency were not statistically different between the two groups.

### Feature Selection

**Figure 1:**
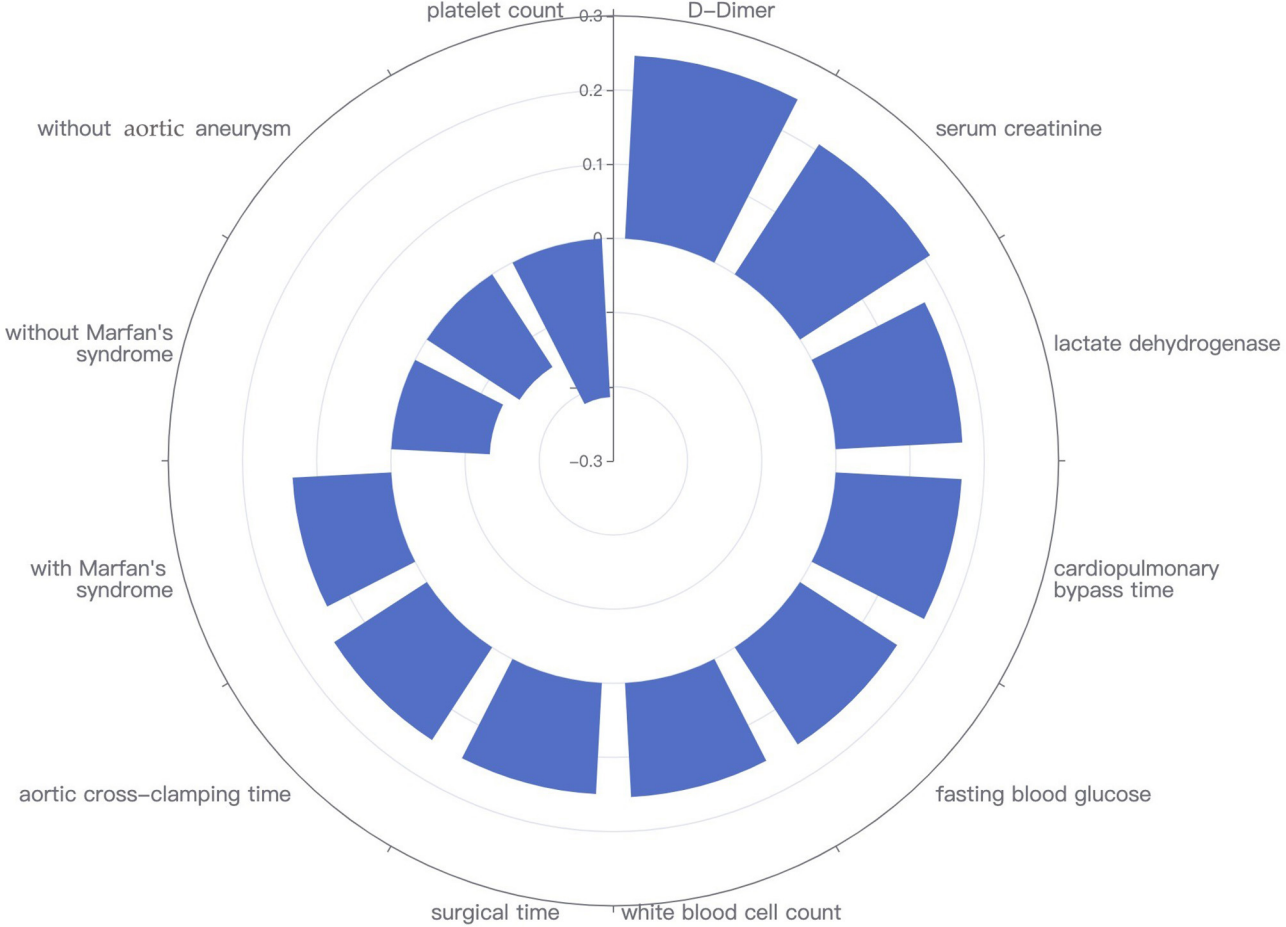
Twelve features selected by Kendall correlation coefficient for models building.

Fourteen features were selected by Kendall correlation coefficient. After excluding two clinically irrelevant variables (venous cannulation position: superior/inferior vena cava and venous cannulation position: right atrium/vena cava), 12 features were eventually extracted to build the models, namely, 9 preoperative and 3 intraoperative features (Figure 1). They were ranked as follows: D-dimer (τ = 0.247), serum creatinine (τ = 0.209), lactate dehydrogenase (τ = 0.171), cardiopulmonary bypass time (τ = 0.170), fasting blood glucose (τ = 0.156), white blood cell count (τ = 0.154), surgical time (τ = 0.150), aortic cross-clamping time (τ = 0.149), with Marfan's syndrome (τ = 0.133), without Marfan's syndrome (τ = −0.133), without aortic aneurysm (τ = −0.149), and platelet count (τ = −0.214).

### Machine Learning Models' Performance for Predicting ICU-LOS

**Figure 2:**
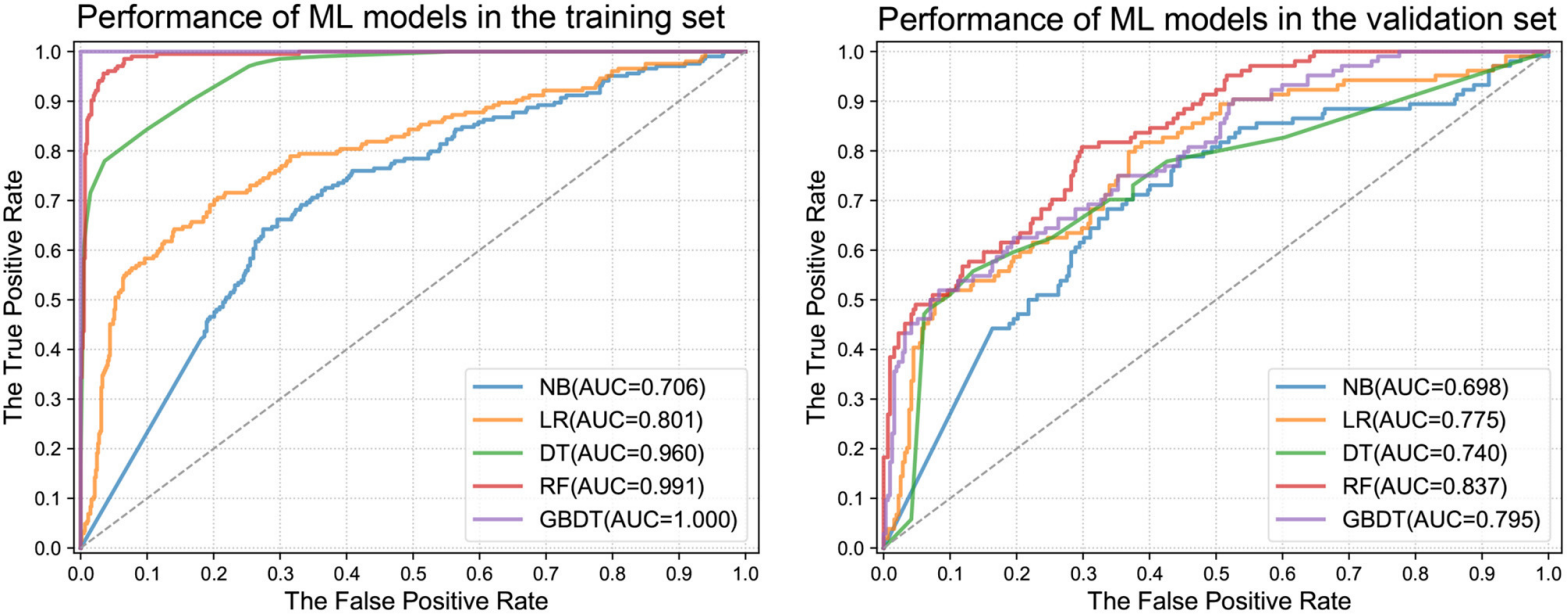
Receiver operating characteristic area under the curves of machine learning models in the training and validation datasets. ML, machine learning; NB, Naive Bayes; LR, Linear Regression; DT, Decision Tree; RF, Random Forest; GBDT, Gradient Boosting Decision Tree; AUC, area under the curve.

**Figure 3:**
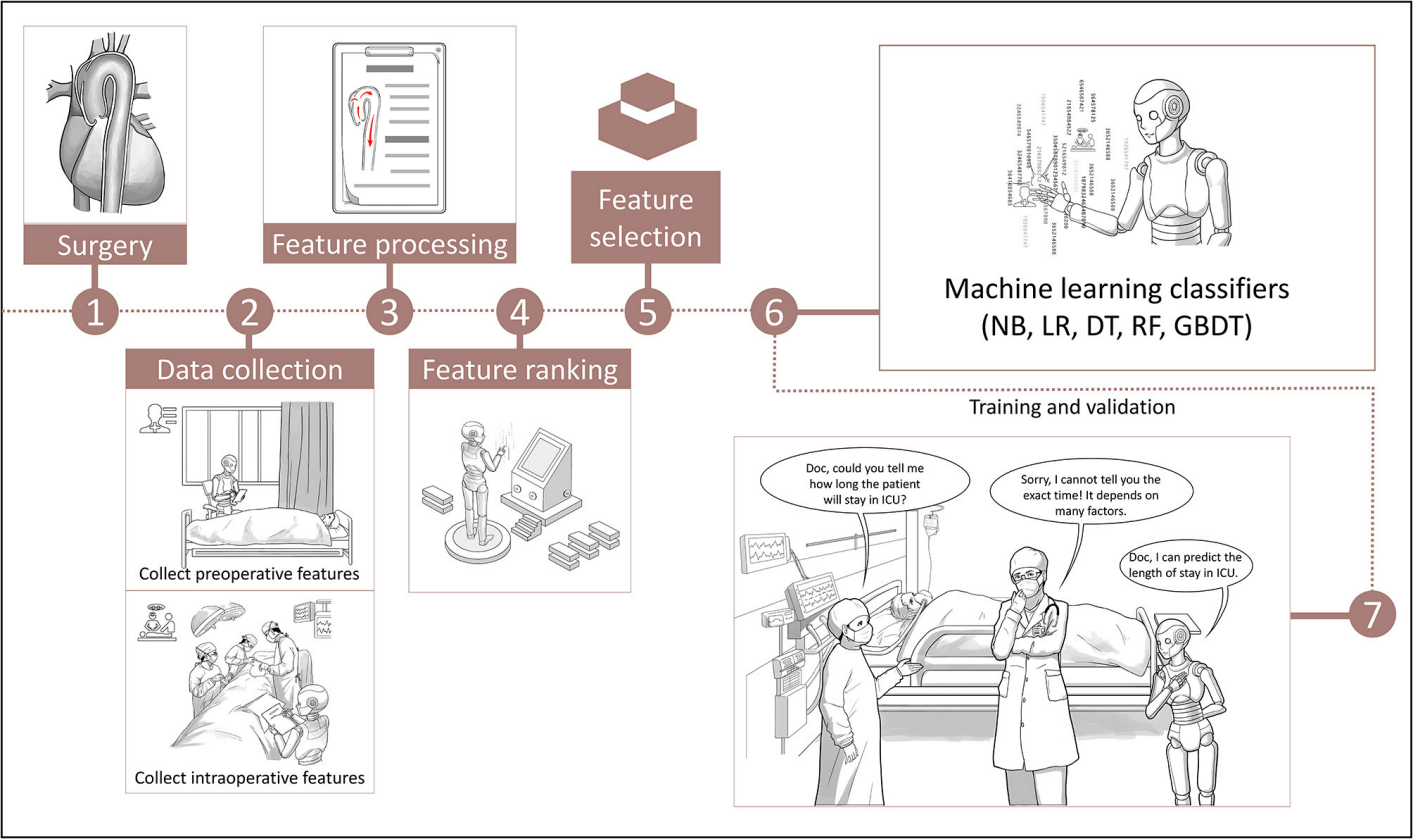
The cartoon flow chart of machine learning models building. NB, Naive Bayes; LR, Linear Regression; DT, Decision Tree; RF, Random Forest; GBDT, Gradient Boosting Decision Tree; ICU, intensive care unit.

The predictive performance of different machine learning models is illustrated in Figure 2. The models had diverse abilities in predicting categorical ICU-LOS. Among the five classifiers, RF achieved the highest performance, with an AUC of 0.991 (95% CI: 0.978–1.000) in the training dataset and 0.837 (95% CI: 0.766–0.908) in the validation dataset. Figure 3 depicts the cartoon of machine learning models building.

## Discussion

The study showed that machine learning classifiers could accurately predict ICU-LOS in patients after acute type A aortic dissection surgery. RF had the highest performance in predicting ICU-LOS. The 12 predictors included in the models were generally readily available at the hospital. D-dimer, serum creatinine, and lactate dehydrogenase were the top three preoperative predictors, and the cardiopulmonary bypass time, surgical time, and aortic cross-clamping time were the top three intraoperative predictors.

The selection of the patients determines the applicability of the models constructed in the respective studies. Although there were models to predict ICU-LOS (6, 9, 18, 19), most of them focused on cardiac surgical patients and thus may not be suitable for aortic dissection surgical patients. The logistic regression was the most common model, with AUCs ranging from 0.60 to 0.84. The cutoff values for predicting ICU-LOS in those models differed greatly, for instance, 24, 55, and 72 h. Compared with the previous regression models, the models were built using the novel machine learning methods and included as many factors that may influence the ICU-LOS as possible. Consequently, the accuracy of the machine learning models could be up to 99%. This study may pave the way for the application of machine learning in the field of aortic dissection and promote further works on this topic.

Identifying the risk factors that significantly affect the ICU-LOS enables making more effective plan to reduce ICU duration (8, 20). Many studies (6, 8–12, 21) have evaluated the risk factors for ICU-LOS after cardiac surgeries, including type of surgery, emergent status, renal dysfunction, creatinine, sex, age, left ventricular function, myocardial infarction, cardiopulmonary bypass time, aortic cross-clamp time, and previous cardiac operation. We found that serum creatinine, lactate dehydrogenase, cardiopulmonary bypass time, and aortic cross-clamp time have been reported previously. Moreover, we identified some new factors, such as Marfan's syndrome and aortic aneurysm. It is not unexpected that Marfan's syndrome and aortic aneurysm were risk factors as both may need more extensive surgery, longer surgical time, and longer aortic cross-clamp time. However, we did not report some factors that were recognized as crucial predictors of prognosis for aortic dissection, such as concomitant malperfusion and preoperative ventilatory support, which may impair the power of prediction model.

It is important to select the final model by using different machine learning methods as each machine learning approach has its strength and weakness for different data forms. In the selection of classification models, we used NB, LR, DT, RF, and GBDT for modeling. The NB and LR models require high independence of features, but most of our features are dummy variables with a correlation with each other. Thus, the two models had poor performance in predicting ICU-LOS. The DT model has the advantages of high classification accuracy, simple mode generation, and good robustness to noisy data. RF is a supervised learning algorithm, which is an integrated learning algorithm based on the DT model. It shows excellent performance in classification and regression. The GBDT model is a result of integrated learning of DT, which incorporates the benefits of multiple machine learners. We found that these three models had satisfactory predictive performance, and RF was the best. These results confirm the explorative nature of the machine learning process that requires iterative and explorative experiments in order to discover the model design that can achieve the target accuracy for a specific problem.

However, there are some limitations that should be acknowledged. First, the data were analyzed retrospectively using electronic medical records not originally designed for the analyses performed. However, this authenticates our analysis; it confirms its utility in a real-world clinical setting. Second, this model was not externally validated, which may be not reflective and may also restrict generalizability. Further studies are warranted to address the viability of this model. Finally, the complexity and abstractness of machine learning models make it difficult to explain, which may hinder its reproducibility and clinical application. Advanced techniques are expected to be developed to make the content of machine learning easier to be understood.

Machine learning for big data analysis has revolutionized the traditional way of conducting cardiovascular disease research (22). Machine learning provides an innovative approach to data analysis and imaging interpretation beyond what is provided by conventional statistics. The ability to automatically handle large multidimensional and multivariate data could ultimately expose novel associations between specific features and outcome, and identify trends and patterns that would not be apparent to investigators. With the growing amount of patient data and the rapid implementation of automated algorithms in other fields of medicine, artificial intelligence will shortly become an indispensable part of clinical medicine (23).

## Conclusions

We developed and validated machine learning models to predict the ICU-LOS in patients with acute type A aortic dissection. The performance of RF was the best with accuracy based on AUCs in the training dataset of 0.991 and the validation dataset of 0.837. The 12 predictors required for calculation of the ICU-LOS are generally readily available at the hospital. However, external validation is necessary to address the generalizability of the model.

## Data Availability Statement

The original contributions presented in the study are included in the article/Supplementary Material, further inquiries can be directed to the corresponding author/s.

## Ethics Statement

The studies involving human participants were reviewed and approved by Ethics Committee of the First Affiliated Hospital of Jinan University. Written informed consent for participation was not required for this study in accordance with the national legislation and the institutional requirements.

## Author Contributions

QC, BZ, and JY contributed to the conception and design of the study, the analysis and interpretation of data, and the work draft. XM, LZ, FW, JF, and WH participated in the data extraction and analysis. ML and ZC designed figures and tables. RF and SZ offered guidance in study design and revised the article critically for important intellectual content. All authors read and approved the final version of the manuscript.

## Conflict of Interest

The authors declare that the research was conducted in the absence of any commercial or financial relationships that could be construed as a potential conflict of interest.
