# Supplementary material for: Predicting Intensive Care Unit Length of Stay After Acute Type A Aortic Dissection Surgery Using Machine Learning
Source: Front Cardiovasc Med. 2021 Jul 12;8:675431. doi: 10.3389/fcvm.2021.675431 (PMC8310912; doi:10.3389/fcvm.2021.675431)
Supplement: Supplementary file 1 [file Table_1.docx]

**Supplemental table 1. Comparison of baseline characteristics between the training and validation datasets**

| **Predictor variables** | **Overall**  **(n = 353)** | **Training dataset**  **(n = 247)** | **Validation dataset**  **(n = 106)** | **P value^※^** |
| --- | --- | --- | --- | --- |
| **Preoperative features** | | | | |
| **Basic information of patients** |  |  |  |  |
| Male | 307 (87.0) | 215 (87.0) | 92 (86.8) | 0.95 |
| Pain | 321 (90.9) | 227 (91.9) | 94 (88.7) | 0.33 |
| Cardiac arrest | 6 (1.7) | 4 (1.6) | 2 (1.9) | 0.86 |
| Emergency treatments | 128 (36.3) | 93 (37.7) | 35 (33.0) | 0.41 |
| **Basic vital signs** |  |  |  |  |
| SBP (mmHg) | 114.70±26.96 | 114.92±26.66 | 114.21±27.77 | 0.82 |
| DBP (mmHg) | 86.18±26.78 | 85.48±26.04 | 87.80±28.49 | 0.46 |
| Body temperature (℃) | 36.64±0.43 | 36.64±0.44 | 36.65±0.40 | 0.89 |
| Pulse (bpm) | 80.91±11.50 | 80.49±10.89 | 81.87±12.81 | 0.30 |
| Respiratory frequency (bpm) | 19.90±1.90 | 19.76±1.38 | 20.23±2.74 | 0.03 |
| BMI | 23.53±3.51 | 23.37±3.47 | 23.90±3.58 | 0.19 |
| **ECG** |  |  |  |  |
| Coronary artery involvement | 52 (14.7) | 34 (13.8) | 18 (17.0) | 0.44 |
| **UCG** |  |  |  |  |
| EF (%) | 64.40±6.62 | 64.37±6.85 | 64.49±6.09 | 0.87 |
| IDAA (mm) | 44.76±8.67 | 44.578.76 | 45.22±8.48 | 0.52 |
| AORD (mm) | 37.14±7.75 | 37.16±7.90 | 37.08±7.42 | 0.94 |
| PAH |  |  |  |  |
| Mild | 17 (4.8) | 15 (6.1) | 2 (1.9) | 0.27 |
| Moderate | 6 (1.7) | 5 (2.0) | 1 (0.9) |  |
| Mitral stenosis | 23 (6.5) | 14 (5.7) | 9 (8.5) | 0.33 |
| LVDD (mm) | 171 (48.4) | 127 (51.4) | 44 (41.5) | 0.31 |
| Cardiac tamponade | 20 (5.7) | 14 (5.7) | 6 (5.7) | 1.00 |
| Mitral valvulization of the aorta | 4 (1.1) | 2 (0.8) | 2 (1.9) | 0.38 |
| Pleural effusion | 126 (35.7) | 92 (37.2) | 34 (32.1) | 0.35 |
| **Biochemical examination** |  |  |  |  |
| WBC (×10^9/L) | 12.47±4.58 | 12.56±4.80 | 12.26±4.01 | 0.58 |
| RBC (×10^12/L) | 4.38 (3.88, 4.79) | 4.38 (3.84, 4.80) | 4.43 (3.97, 4.79) | 0.06 |
| Hemoglobin (g/L) | 125.37±24.30 | 125.40±25.90 | 125.28±20.18 | 0.97 |
| Platelet count (×10^9/L) | 212.08±103.69 | 213.49±111.20 | 208.79±84.03 | 0.70 |
| ALT (U/L) | 45.00±150.11 | 41.38±74.70 | 53.43±249.72 | 0.49 |
| AST (U/L) | 56.68±268.00 | 48.59±130.15 | 75.51±447.86 | 0.39 |
| Cr (umol/L) | 106.09±55.09 | 104.52±50.65 | 109.75±64.39 | 0.41 |
| GFR (ml/min) | 84.15±16.54 | 83.20±15.72 | 86.35±18.20 | 0.10 |
| APTT (s) | 40.99±8.85 | 40.72±8.45 | 41.60±9.75 | 0.39 |
| INR | 1.14 (1.08, 1.23) | 1.15 (1.06, 1.22) | 1.14 (1.08, 1.26) | 0.09 |
| LDH (U/L) | 223.00 (185.50, 292.00) | 225.00 (183.00, 293.00) | 217.50 (188.75, 291.50) | 0.30 |
| FBG (mmol/L) | 7.57±3.45 | 7.75±3.88 | 7.16±2.09 | 0.14 |
| HBALC (%) | 5.62±0.29 | 5.63±0.29 | 5.61±0.30 | 0.70 |
| D-Dimer (ng/ml) | 10,319.82±12,731.36 | 10,757.99±14,356.38 | 9,298.79±7,681.05 | 0.32 |
| BUN (mmol/L) | 8.47±24.69 | 9.10±29.31 | 6.99±5.25 | 0.46 |
| Albumin (g/L) | 37.87±22.34 | 38.54±26.02 | 36.32±9.14 | 0.39 |
| CK-MB (U/L) | 19.97±58.10 | 20.24±66.24 | 19.34±32.21 | 0.89 |
| **Vascular conditions** |  |  |  |  |
| Dissection aneurysm classification |  |  |  |  |
| Type C | 284 (80.5) | 192 (77.7) | 92 (86.8) | 0.05 |
| Type S | 69 (19.5) | 55 (22.3) | 14 (13.2) |  |
| Intramural hematoma | 15 (4.2) | 12 (4.9) | 3 (2.8) | 0.39 |
| Aortic aneurysm | 22 (6.2) | 19 (7.7) | 3 (2.8) | 0.08 |
| Type A aortic dissection staging |  |  |  |  |
| Acute | 311 (88.1) | 217 (87.9) | 94 (88.7) | 0.30 |
| Subacute | 20 (5.7) | 12 (4.9) | 8 (7.5) |  |
| Chronic | 22 (6.2) | 18 (7.3) | 4 (3.8) |  |
| **Previous history** |  |  |  |  |
| Drinking | 16 (4.5) | 12 (4.9) | 4 (3.8) | 0.65 |
| Smoking | 58 (16.4) | 41 (16.6) | 17 (16.0) | 0.90 |
| Coronary stent implantation | 8 (2.3) | 5 (2.0) | 3 (2.8) | 0.64 |
| Valvular disease | 34 (9.6) | 24 (9.7) | 10 (9.4) | 0.93 |
| PCI | 6 (1.7) | 4 (1.6) | 2 (1.9) | 0.86 |
| Circulation system disease | 80 (22.7) | 55 (22.3) | 25 (23.6) | 0.79 |
| Endocrine and metabolic systems disease | 13 (3.7) | 8 (3.2) | 5 (4.7) | 0.50 |
| Respiratory system disease | 15 (4.2) | 13 (5.3) | 2 (1.9) | 0.15 |
| Nervous system disease | 14 (4.0) | 10 (4.0) | 4 (3.8) | 0.90 |
| Digestive system disease | 26 (7.4) | 19 (7.7) | 7 (6.6) | 0.72 |
| Urinary system disease | 20 (5.7) | 11 (4.5) | 9 (8.5) | 0.13 |
| Immune system disease | 6 (1.7) | 4 (1.6) | 2 (1.9) | 0.86 |
| Hypertension | 211 (59.8) | 150 (60.7) | 61 (57.5) | 0.58 |
| Cerebral infarction | 14 (4.0) | 11 (4.5) | 3 (2.8) | 0.47 |
| Diabetes | 10 (2.8) | 7 (2.8) | 3 (2.8) | 1.00 |
| Marfan's syndrome | 14 (4.0) | 10 (4.0) | 4 (3.8) | 0.90 |
| CHD | 26 (7.4) | 16 (6.5) | 10 (9.4) | 0.33 |
| **Intraoperative features** | | | | |
| **Intraoperative observation** |  |  |  |  |
| Coronary artery involvement |  |  |  |  |
| Left | 15 (4.2) | 9 (3.6) | 6 (5.7) | 0.25 |
| Right | 88 (24.9) | 59 (23.9) | 29 (27.4) |  |
| Bilateral | 7 (2.0) | 7 (2.8) | 0 (0) |  |
| The location of the rupture |  |  |  |  |
| Aortic root | 55 (15.6) | 38 (15.4) | 17 (16.0) | 0.98 |
| Ascending aorta | 126 (35.7) | 87 (35.2) | 39 (36.8) |  |
| Aortic arch | 105 (29.7) | 74 (30.0) | 31 (29.2) |  |
| Descending thoracic aorta | 15 (4.2) | 10 (4.0) | 5 (4.7) |  |
| Others | 52 (14.7) | 38 (15.4) | 14 (13.2) |  |
| **Type of surgery** |  |  |  |  |
| Cardiac valvular surgery | 78 (22.1) | 52 (21.1) | 26 (24.5) | 0.47 |
| Aortic root surgery |  |  |  |  |
| Complete aortic arch replacement with retained brachiocephalic vessels | 126 (35.7) | 85 (34.4) | 41 (38.7) | 0.89 |
| Complete aortic arch replacement with four branching vessels | 213 (60.3) | 152 (61.5) | 61 (57.5) |  |
| Partial aortic arch replacement | 11 (3.1) | 8 (3.2) | 3 (2.8) |  |
| Descending thoracic aorta surgery | 14 (4.0) | 10 (4.0) | 4 (3.8) | 0.90 |
| Coronary artery bypass surgery | 22 (6.2) | 14 (5.7) | 8 (7.5) | 0.50 |
| **Intraoperative operating** |  |  |  |  |
| Nasopharyngeal temperature at cessation of circulation | 23.89±3.08 | 23.99±2.78 | 23.64±3.70 | 0.32 |
| Extracorporeal circulation temperature control |  |  |  |  |
| 20-25℃ | 296 (83.9) | 206 (83.4) | 90 (84.9) | 0.62 |
| 15-20℃ | 7 (2.0) | 4 (1.6) | 3 (2.8) |  |
| 26-31℃ | 50 (14.2) | 37 (15.0) | 13 (12.3) |  |
| Arterial cannulation position |  |  |  |  |
| Right axillary artery | 256 (72.5) | 180 (72.9) | 76 (71.7) | 0.87 |
| Right femoral artery | 85 (24.1) | 58 (23.5) | 27 (25.5) |  |
| Left femoral artery | 12 (3.4) | 9 (3.6) | 3 (2.8) |  |
| Venous cannulation position |  |  |  |  |
| Vena cava | 194 (55.0) | 135 (54.7) | 59 (55.7) | 0.43 |
| Right atrium/vena cava | 151 (42.8) | 108 (43.7) | 43 (40.6) |  |
| Femoral vein | 8 (2.3) | 4 (1.6) | 4 (3.8) |  |
| **Time** |  |  |  |  |
| Emergency operation |  |  |  |  |
| <6h | 111 (31.4) | 83 (33.6) | 28 (26.4) | 0.41 |
| 6-24h | 105 (29.7) | 74 (30.0) | 31 (29.2) |  |
| 24-48h | 39 (11.0) | 24 (9.7) | 15 (14.2) |  |
| >48h | 98 (27.8) | 66 (26.7) | 32 (30.2) |  |
| Surgical time | 512.41±124.82 | 510.33±120.63 | 517.25±134.56 | 0.63 |
| Cardiopulmonary bypass time | 232.92±58.89 | 233.18±58.23 | 232.31±60.68 | 0.90 |
| Aortic cross-clamping time | 131.21±36.86 | 130.82±38.06 | 132.11±34.06 | 0.76 |
| Body circulation arrest time | 22.61±18.14 | 22.65±17.89 | 22.52±18.81 | 0.95 |

**Note: ^※^**Comparisons between the different groups were performed by using the chi-squared test or Fisher’s exact test when variables were categorical and the 2-tailed Student’s t-test for normally distributed continuous variables, whereas the Mann–Whitney U-test was used when variables were not normal for means comparison.

**Abbreviations:** bpm = beat per minute; UCG = ultrasonic cardiogram; ECG = electrocardiograph; CHD = coronary heart disease; PAH = pulmonary arterial hypertension; EF = ejection fraction; IDAA = Inner diameter of ascending aorta; AORD = aortic root dimension; LVDD = left ventricular diastolic dysfunction; SBP = systolic blood pressure; DBP = diastolic blood pressure; BMI = body mass index; WBC = white blood cell count; RBC = red blood cell count; ALT = alanine aminotransferase; AST = aspartate aminotransferase; Cr = creatinine; GFR = glomerular filtration rate; APTT = activated partial thromboplastin time; INR = international normalized ratio; LDH = lactic dehydrogenase; FBG = fasting blood glucose; HBALC = glycosylated hemoglobin; BUN = blood urea nitrogen; CK-MB = Creatine Kinase-MB.
